# Effect of probiotic chewing tablets on early childhood caries – a randomized controlled trial

**DOI:** 10.1186/s12903-015-0096-5

**Published:** 2015-09-24

**Authors:** Trifa Hedayati-Hajikand, Ulrika Lundberg, Catarina Eldh, Svante Twetman

**Affiliations:** Public Dental Care Service, Scania Region, Sweden; Postgraduate Dental Education Center, Region Örebro län, Sweden; Department of Odontology, Faculty of Health and Medical Sciences, University of Copenhagen, Nørre Allé 20, 2200 Copenhagen, Denmark

**Keywords:** Chewing tablets, Preschool children, Prevention, Probiotics

## Abstract

**Background:**

To evaluate the effect of probiotic chewing tablets on early childhood caries development in preschool children living in a low socioeconomic multicultural area.

**Methods:**

The investigation employed a randomized double-blind placebo-controlled design. The study group consisted of 138 healthy 2-3-year-old children that were consecutively recruited after informed parental consent. After enrollment, they were randomized to a test or a placebo group. The parents of the test group were instructed to give their child one chewing tablet per day containing three strains of live probiotic bacteria (ProBiora3®) and the placebo group got identical tablets without bacteria. The duration was one year and the prevalence and increment of initial and manifest caries lesions was examined at baseline and follow-up. All parents were thoroughly instructed to brush the teeth of their off-springs twice daily with fluoride toothpaste.

**Results:**

The groups were balanced at baseline and the attrition rate was 20 %. Around 2/3 of the children in both groups reported an acceptable compliance. The caries increment (Δds) was significantly lower in the test group when compared with the placebo group, 0.2 *vs.* 0.8 (*p* < 0.05). The risk reduction was 0.47 (95 % CI 0.24–0.98) and the number needed to treat close to five. No differences were displayed between the groups concerning presence of visible plaque or bleeding-on-brushing. No side effects were reported.

**Conclusions:**

The results suggested that early childhood caries development could be reduced through administration of these probiotic chewing tablets as adjunct to daily use of fluoride toothpaste in preschool children. Further studies on a possible dose–response relationship seem justified

**Trial registration:**

ClinicalTrials.gov Identifier: NCT01720771. First received: October 31, 2012.

**Electronic supplementary material:**

The online version of this article (doi:10.1186/s12903-015-0096-5) contains supplementary material, which is available to authorized users.

## Background

Probiotics are defined as live microorganisms that confer a health benefit on the host when administrated in appropriate amounts [1]. During the last decade, the use of such beneficial bacteria has gained interest within the dental research community with focus on caries development, periodontal health and halitosis [2–4]. For caries prevention and root caries arrest, some studies have provided encouraging results in children and adults [5–8], albeit the quality of evidence still is regarded as insufficient [9]. It is generally thought that exposure to probiotic bacteria early in life may have a greater impact on general and oral health compared to adult regimes [10, 11]. For example, it has been shown that administration of probiotic drops containing strains of *Lactobacillus reuteri* during the first year of life had an impact on caries prevalence and frequency at the age of 9 years [12] Others have however presented contrasting results [13]. As early childhood caries is one of the most serious and costly health conditions among young children [14], it seems important to investigate novel self-administrated preventive strategies that could be added to existing evidence-based recommendations [15]. The aim of the present study was therefore to evaluate the effect of a probiotic chewing tablet, given as a daily supplement after fluoride toothpaste use, on the development of early childhood caries in preschool children living in a multicultural low-socioeconomic area. The null hypothesis was that the caries increment would not differ from that of a control group with similar intake of placebo tablets.

## Methods

### Study group

**Fig. 1:**
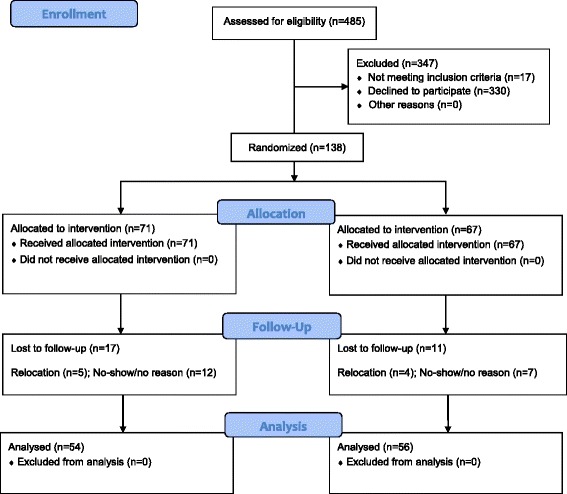
Flow chart of available, eligible and randomized children, indicating the reasons for dropping out

The study group consisted of 138 healthy children, 2–3 years of age that were invited and consecutively enrolled after informed consent from both parents. The inclusion criteria were i) absence of severe chronic disease or allergy, and ii) ability to cooperate at a dental examination. Exclusion criteria were a) ongoing medication with antibiotics, b) regular use of other probiotic products (dairy products or other supplements), c) caries lesions with a need for extractions and restorative care, and d) mentally or physically disabled children. The families had an immigrant background and were inhabitants in Rosengård, a multi-cultural low socio-economic suburban area of Malmö, Sweden. An interpreter was used to explain the study purpose and its procedures in cases with a language barrier. During the study, 28 children (20.3 %) dropped out and the main reasons were family relocation, within or outside the country, and failure to adhere to the study protocol. Thus, the final evaluation was made on 110 children as detailed in Fig. 1. The enrollment of children started in November 2012 and the final recordings were done in July 2014.

### Study design

The investigation employed a randomized, double-blind, placebo-controlled design with a treatment duration of one year. The project was ethically approved by the regional ethics committee in Lund, Sweden (ref nr: 2011/530) and registered in ClinicalTrials.gov. (NCT01720771). The primary outcome was caries increment and secondary endpoints were presence of plaque and gingival health.

### Intervention

After the baseline examination, the children were randomized to one of two parallel groups, test or placebo, with the aid of computer-generated numbers (Excel randomization tool) that were kept in sealed envelopes to ensure allocation concealment. The children of the test group received probiotic chewing tablets containing not less than a 1 × 10^8^ total CFU ProBiora3® blend of three strains of probiotic bacteria (*S. uberis* KJ2™, *S. oralis* KJ3™, *S. rattus* JH145™). The parents were instructed to give their child one tablet per day after brushing teeth. The tablets (EvoraKids) had a wild cherry flavor and were sweetened with erythritol, a natural low-cariogenic polylol. The placebo group received identical chewing tables without probiotic bacteria and the same instructions. The tablets were packed, coded and supplied by Oragenics Inc., FL, USA. The parents were given tablets for 3 months at the time, and asked to bring back all non-used tablets to the clinical team when picking up the next supply. The compliance was rated as “acceptable” when ≤3 tablets per week were forgotten and as “questionable” when this happened more frequently. In connection with the enrolment, all parents were thoroughly instructed to carry out tooth brushing with a smear layer of fluoridated toothpaste (1,100 ppm) twice a day (morning and evening) throughout the entire study period. The toothpaste was supplied by the investigators and handed out at each visit. The parents were encouraged to immediately report any possible perceived harmful effects to the clinical staff and stop the intake.

### Clinical registrations

The children were clinically examined at baseline and after 1 year by one of two trained and calibrated dental hygienists (UL and CE). Caries was visually scored on surface level as “sound” (no evidence of caries), “initial” (first visual change in enamel), “cavitated” (breakdown of enamel, with or without visible dentine) or “arrested” (hard and smooth surface but whitish, brownish or black) after drying and cleaning and expressed as decayed surfaces (ds). Caries increment (Δds) was calculated as the difference between the follow-up scores and baseline for each individual. No radiographs were exposed. The presence of visible supragingival plaque on the buccal surfaces of the upper front teeth was scored as “yes” or “no”. The gingival condition was evaluated as “bleeding-on-brushing”; the teeth were gently brushed by the dental hygienist with a disposable toothbrush and any bleeding along the gingival margin that appeared within 30 s was scored as “yes”. The parents were asked to complete a questionnaire covering their children’s medical history and ongoing dietary and oral hygiene habits. The calibration was carried out immediately prior to the baseline examination against a specialist in pediatric dentistry (“gold standard”). Thereafter, 12 randomly selected 2-year-old children were examined independently by the two clinicians. There was a perfect inter-examiner agreement for presence of visible plaque and bleeding after brushing while the agreement for the caries scores was rated as good (Kappa value 0.63).

### Statistical methods

All data were processed with the IBM SPSS software (version 21.0, Chicago, IL, USA). The groups were compared with the nonparametric Wilcoxon signed rank test for continuous variables and chi-square tests for the categorical data. The absolute risk reduction (ARR) was expressed as the control event rate minus the experimental even rate and the number needed to treat as 1/ARR. A *p*-value <0.05 was considered statistically significant. The group allocation was unknown both for the parents and the investigators and the code was disclosed after the statistical calculations.

A power calculation was conducted for the primary outcome based on a previous study in preschool children from the same local community [16]. With α set at 0.05 and β = 0.2, it was estimated that approximately 70 subjects in each arm would be needed to disclose a 50 % difference between the two groups. In order to adjust for an expected attrition, the goal was to recruit a total number of 175 subjects.

## Results

The baseline characteristics of the two study groups and the dropouts are presented in Table 1. The test and placebo groups were balanced in all aspects and the dropouts did not differ from those that completed the study. The caries prevalence and increment is shown in Table 2. The 1-year increment (Δds) was significantly lower (*p* < 0.05) in the test group compared with the placebo group and the prevented fraction was 75 %. Based on the caries prevalence, the absolute risk reduction was 22 % and the number needed to treat was close to five. The majority of the new lesions were enamel (initial) lesions but no statistically significant differences between the groups concerning the severity, or arrestment of lesions, were displayed (Table 3). An intention-to-treat analysis based on caries increment revealed a relative risk reduction of 0.47 (95 % CI 0.24–0.98). There were no significant differences between the test and the placebo group regarding the prevalence of visible plaque (29 *vs.* 25 %) or bleeding-on-brushing (13 *vs.* 10 %) at the 1-year follow-up.

**Table 1:** Baseline characteristics of the study group

Variable	Test	Placebo	Dropout	*p*
	*n* = 54	*n* = 56	*n* = 28	
Age (mean, SD)	2.4 (0.4)	2.3 (0.4)	2.5 (0.4)	NS
Sex (boys/girls)	52/48 %	45/55 %	46/54 %	NS
Medical problems^a^, n (%)	17 (31 %)	18 (33 %)	9 (32 %)	NS
Frequent sweet meals, n (%)	14 (25 %)	20 (36 %)	9 (32 %)	NS
Supervised tooth brushing (2/day), n (%)	43 (80 %)	44 (79 %)	22 (78 %)	NS
Supervised tooth brushing (1/day), n (%)	9 (17 %)	8 (15 %)	5 (18 %)	NS
Number of erupted teeth (mean, SD)	18.9 (2.4)	18.9 (2.7)	19.2 (1.9)	NS
Visible plaque (yes), n (%)	18 (34 %)	22 (40 %)	11 (41 %)	NS
Bleeding after brushing (yes)	6 (11 %)	4 (7 %)	3 (11 %)	NS
ds (mean, SD)	0.7 (1.4)	0.7 (1.4)	0.8 (1.7)	NS

^a^examples were asthma, frequent infections with antibiotic treatment, frequent diarrhea

*NS* no statistically significant difference

**Table 2:** Caries prevalence at baseline and 1-year caries increment (Δds)

Variable	Test (*n* = 54)	Placebo (*n* = 56)	*p*
Caries prevalence			
Baseline (ds > 0), n (%)	13 (24 %)	15 (27 %)	NS
Follow-up (ds > 0), n (%)	13 (24 %)	26 (47 %)	<0.05
Caries increment			
Δds (mean, SD)	0.2 (1.2)	0.8 (1.4)	<0.05

*NS* no statistically significant difference

**Table 3:** Distribution of initial, cavitated and arrested caries lesions at follow-up

Variable	Test (*n* = 54)	Placebo (*n* = 56)	*p*
Initial	19 %	34 %	NS
Cavitated	14 %	14 %	NS
Arrested	11 %	7 %	NS

Values in table denote the percentage of ds

*NS* no statistically significant difference

The compliance was graded as acceptable among 72 % of the children in the test group and 69 % in the placebo group. No harmful events were reported during the study period among those that completed the intervention. No permanent restorative treatment or extractions were carried out in any of the participants.

## Discussion

To our knowledge, this is the first clinical study that demonstrated a significant reduction of early childhood caries following daily intake of oral probiotic tablets. Thus, the null hypothesis was rejected. The findings were clearly in agreement with some previous reports on probiotic lactobacilli added to milk and served to preschool children attending in public day care centers [5, 6]. In contrast, Taipale and co-workers [17] failed to affect the caries occurrence in 4-year old children after administration of probiotic bifidobacteria with a pacifier or spoon during the first two years of life. However, the latter study was performed in a low-caries population which was in contrast to the present project. It is well known that there is a strong link between socioeconomic status and oral health [18] and it is a challenge to address and overcome such inequalities, especially among young children. Therefore, any educational, lifestyle or behavior-based initiatives to improve oral health among disadvantaged children are important and likely beneficial for their well-being and quality of life [19]. The most important component of such programs is without doubt daily fluoride exposure [20] but our findings indicate that probiotic therapy can be a valuable adjunct in order to increase the effectiveness of the recommended and established preventive care. It should be noted that over 94 % of the parents reported that they helped their children with tooth brushing with fluoride toothpaste, at least once daily, during the intervention period. The fluoride exposure from diet and water (<0.3 ppm) was however considered as low.

The mechanisms of probiotic action are not fully clear but rely on local events, such as co-aggregation, competitive inhibition and bacteriocin production as well as systemic immune-based avenues [3]. For the caries disease, the local effects seem most relevant and in animal models, a competitive inhibition has previously been shown for *S. rattus* and peroxide production for *S. oralis* and *S. uberis* [21]. Furthermore, systematic reviews have concluded that there is good evidence that probiotic supplements can reduce the levels of mutans streptococci in plaque and saliva [9, 22]. Such reductions have also been demonstrated with the ProBiora3® concept [23, 24]. Our results indicated that the intervention mainly affected the early enamel demineralization rather than the cavitated lesions. With the relatively small sample size and short study period in mind, it would have been interesting to follow the impact of an extended intervention on the caries progression. Notably, no new children in the test group exhibited caries lesions during the study period.

The enrollment of healthy volunteers for this project was far from smooth and only around one fourth of the eligible families gave their consent. Many parents hesitated to give their children “pills” on a daily basis for a period of one year. Furthermore, culture and language barriers did not facilitate the recruitment process. Therefore, a certain selection bias cannot be excluded and the external validity for other high caries populations, or age groups, is unclear. The parents that agreed to participate seemed highly motivated for health-promoting actions and this was illustrated by the acceptable compliance reached among the majority of the participants. The relatively high attrition rate of 20.3 % was not unexpected given the volatile nature of the immigrant population. It can be anticipated that the families that did not show up to collect new tablets failed to follow the study protocol. The number of participants indicated in the initial power calculation was not reached, but the caries activity in this study population was somewhat higher than expected which, in part, saved the study from being underpowered. The fact that no adverse events were reported was encouraging and in accordance with the generally recognized as safe (GRAS) nature of probiotic supplements. Initially, a number of children perceived the tablets as “big” and “hard” and refused to take them. In those cases, the parents were encouraged to “crush” the tablets and give them in smaller pieces and this recommendation was clearly helpful.

## Conclusions

Within the limitations of the present study, the results suggested that early childhood caries development could be reduced through daily administration of these probiotic chewing tablets as adjunct to daily use of fluoride toothpaste in preschool children. Therefore, further studies to confirm these findings and clarify a possible dose–response relationship, as well as health economic issues, seem justified.

## Additional file

Additional file 1:
**CONSORT 2010 checklist of information to include when reporting a randomised trial.** (DOC 217 kb)

## Abbreviations

ARR: Absolute risk reduction
CFU: Colony forming units
CI: Confidence interval
ds: Decayed surfaces
GRAS: Generally recognized as safe
ppm: Part per million
